# Establishment of a TRV-VIGS system in Coix (*Coix lacryma-jobi* L.) for functional study of host–pathogen interactions

**DOI:** 10.3389/fpls.2026.1816009

**Published:** 2026-04-14

**Authors:** Xiaosheng Zhao, Guocui Wang, Fang He, Xiang-Dong Li, Chunli Zhou, Qiuping Liu, Leitao Tan, Qingbei Weng

**Affiliations:** 1 School of Life Sciences, Guizhou Normal University, Gui’an, Guizhou, China; 2 Southwest Guizhou Institute of Agricultural and Forestry Sciences, Xingyi, Guizhou, China; 3 Qiannan Normal University for Nationalities, Duyun, Guizhou, China

**Keywords:** *Coix lacryma-jobi* L., functional genomics, host-pathogen interaction, smut fungi, TRV-VIGS

## Abstract

Coix (*Coix lacryma-jobi* L.) is an important cereal and medicinal crop threatened by smut disease (*Ustilago coicis*). However, the unavailability of rapid and efficient functional genomics tools has impeded progress in understanding gene function and host-pathogen interactions in Coix. Here, we report the first virus-induced gene silencing (VIGS) system for Coix using a Tobacco Rattle Virus (TRV) vector. Using the phytoene desaturase gene (*ClPDS*) as a visual reporter, we optimized vacuum infiltration parameters and discovered that the immunosuppressant methylglyoxal dramatically enhances silencing efficiency. Supplementation of the infiltration buffer with 200 μM methylglyoxal boosted silencing efficiency from 25% to 57%, whereas tenoxicam showed no effect. To validate the TRV-VIGS system for studying plant-pathogen interactions, we first developed a *U. coicis* inoculation protocol for Coix seedlings to evaluate smut disease resistance. We then demonstrated the system’s utility by silencing the host defense regulator *ClNPR1*, which increased susceptibility to *U. coicis*, and by silencing the pathogen effector gene *UcPep1* via host-induced gene silencing (HIGS), which enhanced resistance. These findings provide an effective tool for functional genomics studies in Coix and have significant implications for screening and validating potential gene functions under various stresses.

## Introduction

Coix (*Coix lacryma-jobi* L.), also known as Job’s tears, is an important cereal crop with notable medicinal value, holding a significant place in traditional Chinese medicine and diet (Hua et al., 2023; Yang et al., 2025). *Ustilago coicis*, a biotrophic parasitic fungus belonging to the genus *Ustilago*, systemically infects Coix plants, causing Coix smut disease. This disease damages multiple tissues and organs of Coix, particularly filling infected seeds with dark brown spores, severely threatening Coix yield and quality (Li et al., 2020). Numerous studies indicate that identifying pathogen virulence genes and plant disease resistance and related genes are crucial for guiding the development of resistant varieties and plant disease management (Dodds and Rathjen, 2010; Nejat et al., 2017; Jones et al., 2024). The completion of genome sequencing of the Coix in 2020 (Guo et al., 2020; Kang et al., 2020; Liu et al., 2020) and the *U. coicis* in 2022 (Li et al., 2022) have laid a foundation for understanding gene function in this crop and pathogen. However, efficient tools for studying gene function of host–pathogen interactions in Coix and *U. coicis* remain unreported, limiting the functional genomics research and the genetic improvement of this valuable crop.

Virus-induced gene silencing (VIGS) has emerged as a powerful and rapid reverse genetics tool for plant gene functional studies (Bekele et al., 2019). This process exploits the plant’s antiviral RNAi pathway, and a recombinant virus carrying a host gene fragment triggers siRNA-guided degradation of the target mRNA (Voinnet, 2001; Bologna and Voinnet, 2014; Jagram and Dasgupta, 2023). Various plant viruses have been successfully applied in VIGS systems, among which the Tobacco Rattle Virus (TRV)-based system is particularly popular due to its broad host range (Bachan and Dinesh-Kumar, 2012; Shi et al., 2021). TRV has a bipartite genome comprising two distinct positive-sense single-stranded RNAs, RNA1 (responsible for replication and movement) and RNA2 (which carries the viral coat protein and serves as the backbone for inserting target gene sequences) (Ratcliff et al., 2001). TRV vectors have been successfully applied in a variety of plant species, including dicotyledonous plants such as tomato (Liu et al., 2002), *Arabidopsis* (Burch-Smith et al., 2006), tobacco (Senthil-Kumar and Mysore, 2014), chili pepper (Chung et al., 2004), cotton (Gao et al., 2011), petunia (Chen et al., 2005) and soybean (Deng et al., 2025); as well as monocotyledonous plants like *Miscanthus* (He et al., 2023), gladiolus (Singh et al., 2013), wheat and maize (Zhang et al., 2017). Unlike stable genetic transformation, VIGS is usually convenient to perform and genotype-independent, thus enabling rapid, high-throughput screening and validation of gene functions. A recent study employed TRV-VIGS to screen ethylene-related genes induced by *Corynespora cassiicola* in *Hydrangea macrophylla* (Liu et al., 2025), highlighting the potential of TRV-VIGS for dissecting disease resistance pathways in non-model plants. The NPR1 (NONEXPRESSOR OF PATHOGENESIS-RELATED GENES 1) gene plays a crucial role in plant defense against biotrophic pathogens (Zavaliev and Dong, 2024), while the *Ustilago maydis* effector UmPep1 is critical for suppressing maize immunity (Hemetsberger et al., 2015). Based on these findings, the Coix homologs of ZmNPR1 (designated *ClNPR1*) and UmPep1 in *U. coicis* (UcPep1) were selected as target genes to validate a TRV-VIGS system for studying the Coix-smut fungus interaction.

In monocotyledonous plants, *Agrobacterium* inoculation often triggers a strong defense response (Pitzschke, 2013; Zhang et al., 2013), which may lead to a decrease in the efficiency of *Agrobacterium*-mediated VIGS. A recent study reported that treatment with the immunosuppressant tenoxicam inhibits immune responses against bacterial pathogens in *Arabidopsis* (Ishihama et al., 2021). Similarly, another report showed that infection with a virulent bacterial strain elevates endogenous methylglyoxal levels, which in turn promotes pathogen colonization in the same plant species (Yuan et al., 2025). Thus, we hypothesized that applying immunosuppressants (tenoxicam and methylglyoxal) would transiently suppress host basal immunity, consequently facilitating *Agrobacterium* and TRV infection and increasing VIGS efficiency in Coix. To date, no VIGS system has been reported for this important crop.

Here, using the phytoene desaturase gene (*ClPDS*) as a visual marker, we successfully established a highly efficient vacuum infiltration-based TRV-VIGS protocol. And this protocol was optimized by varying *Agrobacterium* concentration and by applying the immunosuppressants tenoxicam and methylglyoxal. Furthermore, we demonstrated the utility of this system by silencing the host resistance gene *ClNPR1*, which compromised resistance to *U. coicis*, and by silencing the pathogen virulence gene *UcPep1* through host-induced gene silencing (HIGS), which enhanced resistance. This newly developed system provides a valuable platform for large-scale functional genomics studies and will greatly facilitate the dissection of molecular mechanisms underlying disease resistance and other biological processes in Coix.

## Materials and methods

### Isolation of haploid strains of *Ustilago coicis*

*U. coicis* was isolated from smut-infected Coix seeds collected from Xingyi, Guizhou Province. Haploid strains were isolated from the teliospores of *U. coicis*. Briefly, teliospores from diseased Coix seeds were dispersed in sterile water using a sterile needle. The spore suspension was serially diluted to 10³ spores/mL, and 100 μL was spread on YEPS (1% yeast extract, 2% peptone, 2% sucrose, 1.5% agar) plates. Plates were incubated at 25 °C for 2 days. Single colonies were picked, suspended in sterile water, and again serially diluted to 10³ CFU/mL before plating on YEPS. After 2 days at 25 °C, 36 single colonies were randomly selected, inoculated into YEPS liquid medium (1% yeast extract, 2% peptone, 2% sucrose), and shaken at 180 rpm, 25 °C for 18 h. These 36 haploid clones were designated Uc-XY-H1 through Uc-XY-H36. Mating assays were performed by pairing clones sequentially (e.g., Uc-XY-H1 with Uc-XY-H19) on YEPS plates, resulting in 18 mating combinations. After 2 days at 25 °C, the combination of Uc-XY-H6 and Uc-XY-H24 produced filamentous growth indicative of successful mating (**Supplementary Figure 4**). Haploid strains Uc-XY-H6 and Uc-XY-H24 were stored at -80 °C for subsequent inoculation experiments.

### Plant material cultivation and pathogen inoculation

Five Coix varieties (Yizhu No.4, Yizhu No.1, Shiyi No.1, Anyi No.1, and XingrenXiaobaike) were used in this study. Plants were grown in a greenhouse (25/22 °C, 80% relative humidity, 14/10 h light/dark cycle). Ten-day-old seedlings were used for *U. coicis* inoculation. Haploid strains Uc-XY-H6 and Uc-XY-H24 were individually cultured in YEPS liquid medium at 180 rpm, 25 °C for 18 h. Cells were harvested by centrifugation at 5,000 rpm for 10 min and resuspended in infiltration buffer (10 mM MgCl_2_) to an OD600 of 0.5. The two suspensions were mixed at a 1:1 volume ratio. The mixed culture was injected into Coix seedling stems using a 1 mL sterile syringe. Infiltration buffer (10 mM MgCl_2_) served as a mock control. Inoculated plants were returned to the greenhouse (25/22 °C, 80% relative humidity, 14/10 h light/dark) for 4 days before disease assessment. Leaves were collected 5 cm below the injection site for imaging, and relative disease area was analyzed using ImageJ software (ImageJ 1.54, NIH, Bethesda, MD, USA). The pathogen inoculation experiment was performed at least three times, each consisting of a minimum of 10 plants.

### Construction of pTRV2 vectors

The Coix *ClPDS* and *ClNPR1* genes, and the *U. coicis UcPep1* gene were selected for VIGS experiments. These genes were identified using TBtools-II (Chen et al., 2023). Briefly, maize PDS (Zm00001d044558, Li et al., 2021) and NPR1 (NP_001354806.1) sequences were used as queries to search the Coix genome database (https://ngdc.cncb.ac.cn/gwh/Assembly/503/show), yielding Coix PDS (GWHPAAYR013449) and NPR1 (GWHPAAYR045617) information. Similarly, the maize smut *UmPep1* gene (AEK86695.1) was used to search the *U. coicis* genome database (https://www.ncbi.nlm.nih.gov/bioproject/PRJNA793722), identifying the *U. coicis* Pep1 homolog (LXDA03987.1). To construct VIGS vectors, 200–300 bp CDS fragments of each gene were cloned. The Coix *PDS* fragment (from 1258 to 1490 of CDS) was ligated into the Kpn I and BamH I sites of pTRV2 to generate pTRV2-ClPDS. The Coix *ClNPR1* and *U. coicis UcPep1* fragments were ligated into the EcoRI and BamHI sites to generate pTRV2-ClNPR1 and pTRV2-UcPep1, respectively. And the pTRV2-derivative vectors were confirmed by PCR and DNA sequencing (Tsingke Biotechnology Co., Ltd.). All primers used for cloning and sequencing are listed in **Supplementary Table 1**.

### Establishment of the vacuum infiltration TRV-VIGS system in Coix

Seeds of Coix variety Yizhu No.4 were surface-sterilized by soaking in 75% (v/v) ethanol for 1 min, followed by 5 min in 2.5% sodium hypochlorite containing 0.1% Tween 20, and then rinsed thoroughly with sterile deionized water. Sterilized seeds were placed on moist sterile filter paper in Petri dishes and germinated in the dark at 28 °C for 36 h. Seedlings with sprouts approximately 3 mm long were selected for VIGS experiments.

The constructed pTRV2-ClPDS vector was transformed into *Agrobacterium tumefaciens* strain GV3101. *Agrobacterium* was cultured overnight in YEP medium at 28 °C, harvested by centrifugation at 5,000 rpm for 10 min, and resuspended in infiltration buffer (10 mM MES, 10 mM MgCl_2_, 200 µM acetosyringone, pH 5.6). The OD600 was adjusted to 0.7, 0.5, 0.3, and 0.1. *Agrobacterium* cultures containing pTRV1 and the different concentrations of pTRV2-ClPDS were mixed at a 1:1 volume ratio. A mixture of pTRV1 and empty pTRV2 *Agrobacterium* served as a mock control.

Vacuum infiltration was performed as described previously (Zhang et al., 2017). Briefly, 20 sterilized germinated seeds were placed in a 10 mL medical glass vial, submerged in 5 mL of the *Agrobacterium* mixture, and sealed with a rubber stopper. A vacuum of approximately 20 kPa was applied using a 20 mL syringe and maintained for 30 seconds. Seeds in the bacterial suspension were then incubated in the dark at 25 °C for 6 h. After washing off excess *Agrobacterium* with sterile water, seeds were planted in soil and transferred to the greenhouse (25/22 °C, 80% relative humidity, 14/10 h light/dark cycle) for cultivation. The VIGS experiment was repeated at least three times with a minimum of 20 plants per replicate.

### Application of tenoxicam and methylglyoxal

Stock solutions (100 mM) of Tenoxicam (MACKLIN^®^, Shanghai Macklin Biochemical Technology Co., Ltd.) and Methylglyoxal (MACKLIN^®^, Shanghai Macklin Biochemical Technology Co., Ltd.) were prepared using sterile deionized water. These stock solutions were added to the infiltration buffer (10 mM MES, 10 mM MgCl_2_, 200 µM acetosyringone, pH 5.6) to achieve final working concentrations of 0 μM, 100 μM, and 200 μM.

### RNA/DNA extraction and RT-qPCR

Total RNA was extracted from Coix leaves using the Trizol (Invitrogen) method. First-strand cDNA was synthesized from 1 µg of total RNA in a 20 µL reaction system using the HiScript^®^ III 1st Strand cDNA Synthesis Kit (Vazyme, Cat# R312). Genomic DNA was extracted from Coix leaves using the *EasyPure*^®^ Universal Plant Genomic DNA Kit (Transgen, Cat# EE112-01). RT-qPCR was performed using a QuantStudio™ 3 Real-Time PCR System (Thermo Fisher Scientific, Waltham, MA, USA) with the *PerfectStart*^®^ Green qPCR SuperMix (Transgen, Cat# AQ601-01) according to the manufacturers’ instructions. The Coix *Actin* and *EF1a* genes served as the internal reference for detecting *ClPDS* and *ClNPR1* gene expression. The *UcPpi* (peptidylprolyl isomerase) gene was used as the internal reference for detecting *UcPep1* expression. Relative fungal biomass was estimated by normalizing *UcPpi* levels to *ClActin*. All RT-qPCR primers are listed in **Supplementary Table 1**, and Ct values of the RT-qPCR experiments are listed in **Supplementary Table 2**.

### Determination of chlorophyll and carotenoid content

Leaves from 15-day-old TRV-VIGS seedlings were used for chlorophyll and carotenoid content measurement, following a previously described method (Fargasová and Molnárová, 2010). Briefly, 0.1 g of leaf tissue was ground to a fine powder in liquid nitrogen under dim light. The powder was transferred to 5 mL of 96% (v/v) ethanol, mixed thoroughly, and incubated in the dark for at least 3 h (until the tissue became white). After centrifugation at 8000 rpm for 10 min, the supernatant was collected. Absorbance was measured at 665 nm, 649 nm, and 470 nm using a spectrophotometer, with the extraction solvent as a blank. Chlorophyll a, chlorophyll b, and carotenoid contents were calculated using the following equations (Lichtenthaler and Wellburn, 1983):

Chl a = 13.95(A665) – 6.88(A649)
Chl b = 24.96(A649) – 7.32(A665)
Car = [1000(A470) – 2.06(Chl a) – 114.8(Chl b)]/245

### Phylogenetic analysis and multiple sequence alignment

Eleven PDS homologous protein sequences were retrieved from the NCBI database (https://www.ncbi.nlm.nih.gov/). Multiple sequence alignment of ClPDS and its homologous proteins was performed using ClustalW. Phylogenetic analysis was conducted using the maximum likelihood (ML) method in MEGA 11 software (Tamura et al., 2021), with bootstrap support calculated from 1000 replicates and default parameters for all other settings.

### Statistical analysis

Data are presented as mean ± standard deviation (SD). Significant differences were determined using Student’s *t*-test for comparisons between two groups, and one-way ANOVA followed by Tukey’s multiple comparisons test for comparisons among multiple groups. Statistical analyses were performed using GraphPad Prism 8.0 (GraphPad Software, San Diego, CA, USA).

## Results

### Optimization and establishment of a vacuum infiltration TRV-VIGS system in Coix using *ClPDS* as a visual marker

To establish a TRV-VIGS system in Coix, we first identified a suitable visual marker gene. Using the maize *PDS* gene sequence (Zm00001d044558) as a query, we performed BLAST searches against the Coix genome database and successfully identified the Coix *PDS* ortholog, designated as *ClPDS* (GWHPAAYR013449). Phylogenetic analysis and sequence alignment revealed that ClPDS is conserved in Poaceae species (**Supplementary Figure 1**) and shares 87.31% and 89.09% sequence similarity with ZmPDS and SbPDS (PDS gene of *Sorghum bicolor*), respectively (**Supplementary Figure 2**), confirming its evolutionary conservation and suitability as a silencing marker. A 233-bp fragment from a conserved region of *ClPDS* coding sequence was cloned into the pTRV2 vector to generate pTRV2-ClPDS.

**Figure 1:**
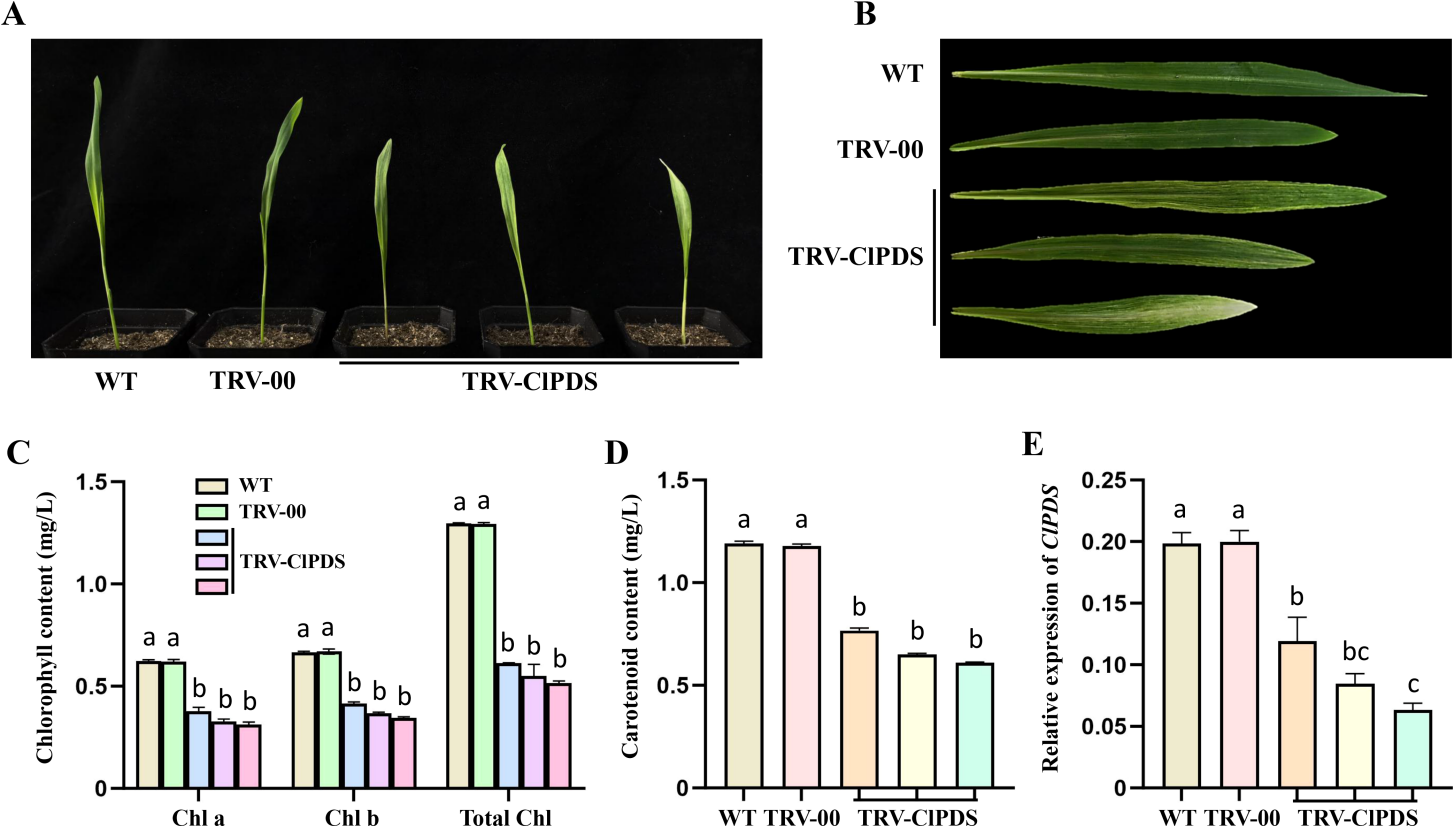
TRV-mediated VIGS of the *ClPDS* gene in *Coix* via vacuum agroinfiltration of germinated seeds. **(A, B)** Phenotypes of entire plants **(A)** and detached leaves **(B)** at 15 days after vacuum infiltration with *Agrobacterium tumefaciens* harboring pTRV1+pTRV2 (TRV-00) or pTRV1+pTRV2-*ClPDS* (TRV-*ClPDS*) to silence the *ClPDS* gene. Wild-type (WT) indicates that only infiltrates *Agrobacterium.***(C, D)** Contents of chlorophyll a, chlorophyll b, total chlorophyll **(C)** and carotenoid content **(D)** in plants at 15 days after TRV-VIGS-mediated silencing of *ClPDS*. **(E)** Relative expression level of *ClPDS* in plants at 15 days after TRV-VIGS-mediated silencing. Data are presented as mean ± SD (n = 3). Similar results were obtained from three independent biological replicates. The expression of *ClPDS* was normalized to the *ClActin* gene. Statistical significance was determined using one-way ANOVA with Tukey’s test (Bars with different letters are significantly different at *p* < 0.05).

**Figure 2:**
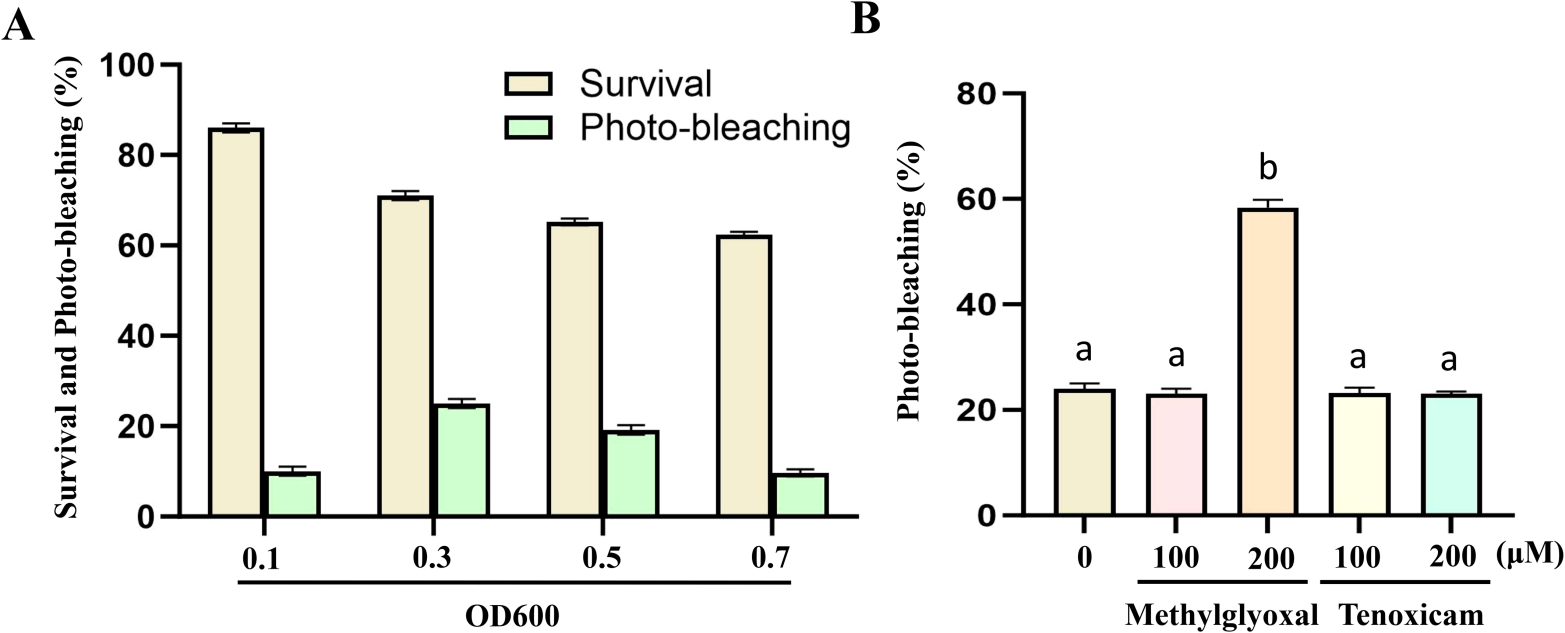
Optimization of TRV-mediated VIGS in *Coix* through *Agrobacterium* concentration and immunosuppressant treatment. **(A)** Effects of different *Agrobacterium* concentrations on survival rate of germination seeds and TRV-VIGS efficiency in Coix. **(B)** Effects of different immunosuppressants (tenoxicam and methylglyoxal) on TRV-VIGS efficiency in Coix at an *Agrobacterium* concentration of OD600 = 0.3. Data are presented as mean ± SD (n = 3). Similar results were obtained from three independent biological replicates. Statistical significance was determined using one-way ANOVA with Tukey’s test (Bars with different letters are significantly different at *p* < 0.05).

We employed a vacuum infiltration method to deliver *Agrobacterium tumefaciens* (strain GV3101) harboring pTRV1 and pTRV2-ClPDS into germinating Coix seeds (**Supplementary Figure 3**). To determine optimal bacterial concentration for maximizing silencing efficiency while maintaining high plant survival, we tested four OD600 values (0.1, 0.3, 0.5, and 0.7) for pTRV2-ClPDS. Germinated Coix seeds (sprout length ~3 mm) were vacuum-infiltrated with *Agrobacterium* suspensions containing pTRV1 and pTRV2-ClPDS (1:1 ratio), with pTRV1+empty pTRV2 as mock control. At 15 days post-infiltration (dpi), plants from all pTRV1+pTRV2-ClPDS treatment groups exhibited visible photobleaching in newly emerged leaves, while control plants remained completely green (**Figures 1A, B**). The highest VIGS efficiency (25%), coupled with a high plant survival rate (71%), was achieved at an OD600 of 0.3 (**Figure 2A**). Biochemical analysis revealed that the photobleaching was accompanied by a significant reduction in both chlorophyll and carotenoid contents in the pTRV1+pTRV2-ClPDS treated plants compared to the control (**Figures 1C, D**). Consistently, RT-qPCR analysis confirmed that *ClPDS* transcript levels were significantly reduced by 40-60% in the photobleached tissues (**Figure 1E**, **Supplementary Figure 6**), directly linking the visual phenotype to gene silencing.

### Enhancing TRV-VIGS efficiency with the immunosuppressant methylglyoxal

To further improve silencing efficiency, we investigated the effect of adding the immunosuppressants tenoxicam and methylglyoxal to the infiltration buffer. Using the optimal *Agrobacterium* concentration (OD600 = 0.3 for pTRV2-ClPDS), we supplemented the infiltration buffer with 0, 100, or 200 µM of each compound. At 15 dpi, the addition of 200 µM methylglyoxal significantly enhanced the VIGS efficiency to 57%, compared to 25% in the control without any additive (**Figure 2B**). In contrast, tenoxicam treatment did not markedly improve silencing efficiency at either concentration tested (**Figure 2B**). Based on these results, the optimized TRV-VIGS protocol for Coix was defined as vacuum infiltration of germinated seeds with an *Agrobacterium* mixture (pTRV1 and pTRV2-derivative, 1:1 ratio) at a final OD600 of 0.3 for the pTRV2-derivative, with the infiltration buffer supplemented with 200 µM methylglyoxal.

### Establishment of a reliable *Ustilago coicis* inoculation system

**Figure 3:**
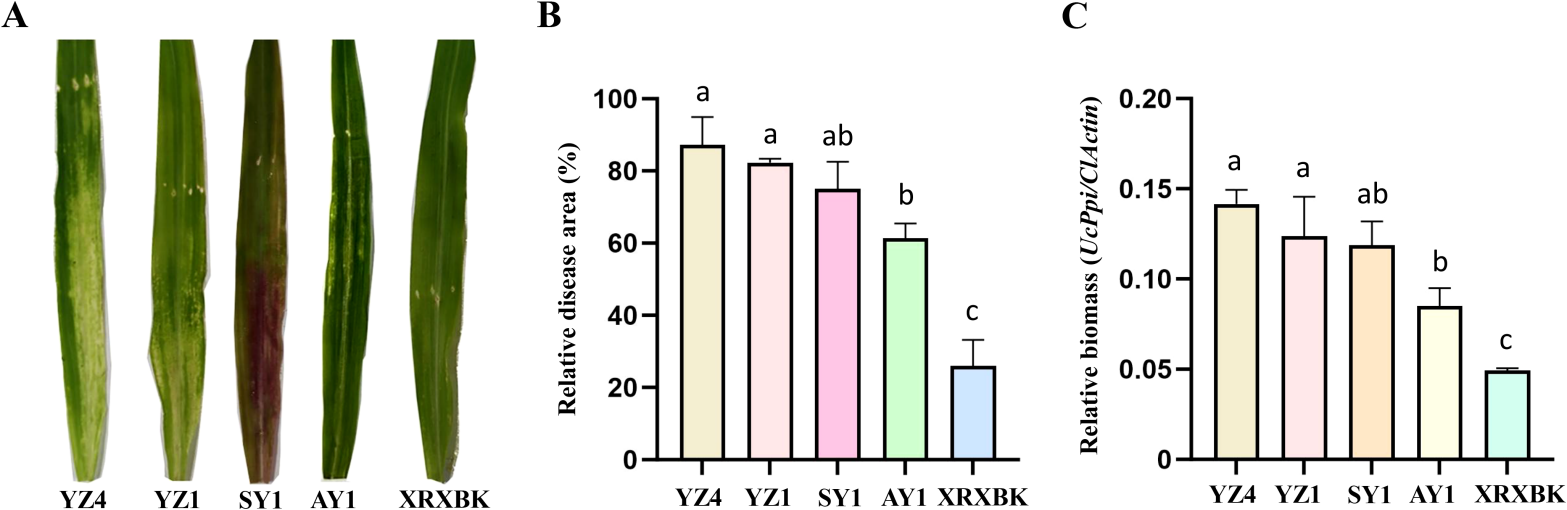
Inoculation and evaluation of *U. coicis* resistance in different Coix cultivars.**(A–C)** Phenotypes **(A)**, relative disease area **(B)**, and relative fungal biomass **(C)** of five Coix varieties, YZ4 (Yizhu No.4), YZ1 (Yizhu No.1), SY1 (Shiyi No.1), AY1 (Anyi No.1), and XRXBK (XingrenXiaobaike), at 4 days post-inoculation with *U. coicis*. Data are presented as mean ± SD (n = 10). Similar results were obtained from three independent biological replicates. Relative disease area was analyzed using ImageJ software (Image J 1.54). Relative fungal biomass was estimated by normalizing *UcPpi* levels to *ClActin*. Statistical significance was determined using one-way ANOVA with Tukey’s test (Bars with different letters are significantly different at *p* < 0.05).

To validate the TRV-VIGS system in plant-pathogen interaction studies, we first established a reliable *U. coicis* infection system. From teliospores collected from smut-infected Coix seeds, we isolated 36 haploid clones through serial dilution. Mating assays identified one compatible pair, Uc-XY-H6 and Uc-XY-H24, which produced characteristic filamentous growth indicating successful mating (**Supplementary Figure 4**). These two strains were selected for all inoculation experiments. We evaluated five Coix varieties (Yizhu No.4, Yizhu No.1, Shiyi No.1, Anyi No.1, and XingrenXiaobaike) for susceptibility to *U. coicis*. Ten-day-old seedlings were syringe-inoculated with a mixed suspension of Uc-XY-H6 and Uc-XY-H24 (two compatible mating-type strains), and by 4 dpi, all varieties developed characteristic lesions (**Figure 3A**). Furthermore, quantitative analysis of relative disease lesion area and fungal biomass revealed that Yizhu No.4 exhibited the largest lesion area (90.1 ± 3.9%) and highest fungal accumulation (relative biomass = 0.14 ± 0.01), indicating high susceptibility (**Figures 3B, C**). In contrast, XingrenXiaobaike showed the greatest resistance of the five *Coix* varieties examined (**Figures 3B, C**). Based on consistent susceptibility and robust growth, XingrenXiaobaike was selected for subsequent VIGS-based functional studies.

### Functional validation of the TRV-VIGS system by silencing host (*ClNPR1*) and pathogen (*UcPep1*) genes

**Figure 4:**
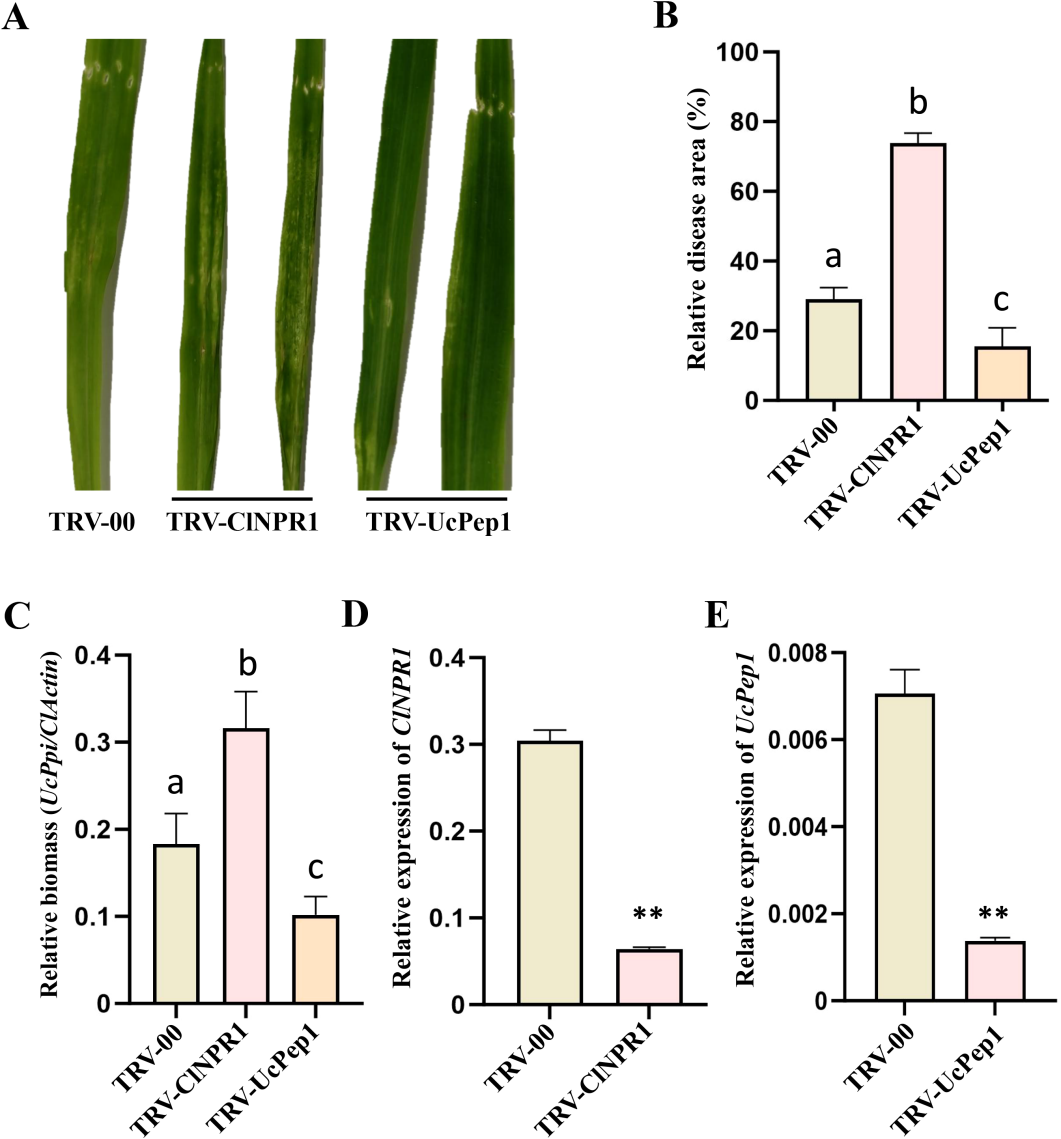
Evaluation of host *ClNPR1* and fungal *UcPep1* roles in smut disease response via TRV-VIGS system in Coix. **(A–E)** Phenotypes **(A)**, relative disease area **(B)**, relative fungal biomass **(C)**, relative expression levels of *ClNPR1***(D)** and *UcPep1***(E)** of TRV-VIGS-silenced *ClNPR1* (TRV-*ClNPR1*) and *UcPep1* (TRV-*UcPep1*) plants 4 days post-inoculation with *U. coicis*. Data are presented as mean ± SD (n = 3). Similar results were obtained from three independent biological replicates. The expression of *ClNPR1* was normalized to the *ClActin* gene; *UcPep1* expression was normalized to the fungal reference gene *UcPpi*. Relative disease area was analyzed using ImageJ software (Image J 1.54). Relative fungal biomass was estimated by normalizing *UcPpi* levels to *ClActin*. Statistical significance was determined using Student’s *t*-test (**p < 0.01) and one-way ANOVA with Tukey’s test (Bars with different letters are significantly different at *p* < 0.05).

To demonstrate the application of the optimized TRV-VIGS system in studying Coix-*U. coicis* interactions, we targeted two genes of interest: the host defense regulator *ClNPR1* and the candidate pathogen effector gene *UcPep1*, a homolog of the *U. maydis* effector Pep1. Coix seeds were vacuum-infiltrated with *Agrobacterium* cultures containing either pTRV1+pTRV2 (control), pTRV1+pTRV2-ClNPR1, or pTRV1+pTRV2-UcPep1, using our optimized protocol with 200 µM methylglyoxal. Ten days post-infiltration, the seedlings were inoculated with a pathogenic mix of *U. coicis* (Uc-XY-H6 and Uc-XY-H24), and disease symptoms were assessed at 4 dpi. Disease assessment revealed opposing phenotypes between treatments (**Figure 4A**). *ClNPR1*-silenced plants exhibited significantly enhanced susceptibility, with lesion area (75.2 ± 3%) approximately 3-fold larger than controls (24.4 ± 1.2%) and fungal biomass (relative biomass = 0.32 ± 0.04) increased approximately 1.8-fold (**Figures 4B, C**). Conversely, *UcPep1*-silenced plants displayed enhanced resistance, with lesion area reduced by approximately 50% and fungal biomass decreased by approximately 40% (**Figures 4B, C**). Furthermore, RT-qPCR confirmed significant downregulation of *ClNPR1/UcPep1*expression in the pTRV1+pTRV2-ClNPR1/pTRV1+pTRV2-UcPep1 group (**Figures 4D, E**). These results indicate that *ClNPR1* functions as a positive regulator of defense against *U. coicis*, while *UcPep1* is a critical pathogenicity factor.

To exclude potential confounding effects of methylglyoxal, we treated plants with 200 μM methylglyoxal alone (without *Agrobacterium*) and inoculated with *U. coicis* at 10 days post-treatment. Disease assessment revealed no significant difference in lesion area or fungal biomass between methylglyoxal-treated and untreated controls (**Supplementary Figure 5**), confirming that observed phenotypes are specifically attributable to gene silencing. Together, these results demonstrate that our optimized TRV-VIGS system is an effective tool for functional analysis of both host and pathogen genes in the Coix-*U. coicis* interaction, establishing a foundation for future high-throughput screening of genes involved in disease resistance and other agronomically important traits in Coix.

## Discussion

Coix represents an important dual-purpose crop with both medicinal and nutritional value. Although its genome was sequenced in 2020 (Guo et al., 2020; Kang et al., 2020; Liu et al., 2020), functional genomic studies have lagged due to the lack of efficient high-throughput tools for gene validation. Virus-induced gene silencing (VIGS) offers a rapid and scalable approach for transient gene knockdown and has been widely applied across many plant species (Bachan and Dinesh-Kumar, 2012; Shi et al., 2021). And TRV has become the most widely adopted viral vector for gene silencing in plants among the nearly 40 viral systems, owing to its broad host range and high efficiency (Tian et al., 2014; Shi et al., 2021). Nevertheless, establishing efficient VIGS systems in non-model plants, especially medicinal species, remains difficult may due to complex secondary metabolites. In this study, we developed a Tobacco rattle virus (TRV)-based VIGS system for germinating Coix seeds by optimizing *Agrobacterium* concentration and applying a plant immunity suppressor. To our knowledge, this is the first successful TRV-VIGS system reported in Coix.

The establishment of VIGS in Coix faced several challenges in present study. Even under optimized conditions (OD600 = 0.3, 30-second vacuum infiltration, 6-hour co-incubation), the initial silencing efficiency for the *PDS* marker gene was only 25%, considerably lower than the 76–90% efficiencies reported in other monocots such as *Miscanthus* (He et al., 2023), wheat and maize (Zhang et al., 2017) under similar vacuum infiltration protocols. This disparity likely reflects intrinsic biological barriers in Coix. First, Monocotyledonous plants typically exhibit a strong defense response to *Agrobacterium* infection, involving the activation of the mitogen-activated protein kinase (MAPK) cascade and a rapid reactive oxygen species (ROS) burst (Pitzschke, 2013; Zhang et al., 2013). It is plausible that a similar defense mechanism is activated in Coix. Second, and perhaps most significantly, Coix seeds accumulate antimicrobial secondary metabolites, such as coixenolide and various phenolic compounds (Hu et al., 2007; Igbokwe et al., 2022; Mahato et al., 2025; Wang et al., 2016), which may suppress viral vector establishment and systemic spread. Consequently, we attempted to enhance VIGS efficiency of Coix by applying immunity suppressors (methylglyoxal and tenoxicam). The substantial improvement in silencing efficiency achieved by methylglyoxal supplementation (from 25% to 57%) warrants mechanistic consideration. Methylglyoxal is a reactive carbonyl compound endogenously produced through glycolysis and other metabolic pathways (Schalkwijk and Stehouwer, 2020). Recent studies have revealed that methylglyoxal can promote bacterial colonization by scavenging ROS, thereby attenuating the oxidative burst that constitutes an early plant defense response (Yuan et al., 2025). In our system, 200 μM methylglyoxal likely functioned similarly, transiently suppressing *Agrobacterium*-triggered immunity and facilitating more efficient T-DNA transfer and viral RNA accumulation. However, methylglyoxal is a pleiotropic molecule; it can also modulate gene expression, protein glycation, and stress signaling (Kaur et al., 2014). The mechanism by which methylglyoxal enhances VIGS efficiency in Coix remains unclear. It is unknown whether this occurs through direct ROS scavenging, suppression of salicylic acid signaling, or other regulatory effects, which require further molecular analyses. Notably, tenoxicam, which antagonizes salicylic acid pathways in *Arabidopsis* (Ishihama et al., 2021), failed to improve silencing in our system, suggesting that methylglyoxal may act through distinct or additional mechanisms. Regardless, the marked effect of methylglyoxal demonstrates that pharmacological suppression of host immunity represents a promising strategy for overcoming recalcitrance in medicinal plants, potentially extending to *Agrobacterium*-mediated transient expression and stable transformation systems in other challenging species.

Smut disease, caused by *U.coicis*, is a major fungal disease in Coix that significantly reduces yield (Li et al., 2020, 2022). We developed a laboratory-based inoculation system using two compatible mating-type strains (Uc-XY-H6 and Uc-XY-H24); injecting a mixed suspension into 10-day-old seedlings enabled reproducible disease assessment within two weeks. This system was used to evaluate resistance levels, facilitating future studies on host–pathogen interactions and the application of VIGS for screening disease resistance-related genes. Functional validation of the VIGS platform through targeting *ClNPR1* confirmed its biological relevance. NPR1 is a master regulator of salicylic acid-mediated systemic acquired resistance, conserved across diverse plant lineages (Zavaliev and Dong, 2024). In *Arabidopsis*, NPR1 is a transcriptional co-activator that, upon salicylic acid accumulation, monomerizes and translocates to the nucleus to activate defense gene expression via TGA transcription factors (Kumar et al., 2022). In *ClNPR1*-silenced Coix plants, infection with *U. coicis* resulted in enlarged lesions and increased fungal biomass, supporting a conserved role for NPR1 in basal resistance against this biotrophic pathogen. This result also validates that our VIGS system achieves sufficient knockdown to produce discernible phenotypic effects, despite the moderate silencing efficiency. Importantly, control experiments confirmed that methylglyoxal treatment alone did not alter disease responses, excluding the possibility that the observed phenotypes were artifacts of immune suppression rather than specific gene silencing.

The application of host-induced gene silencing (HIGS) to target the *U. coicis Pep1* homolog (*UcPep1*) further demonstrates the versatility of this VIGS platform. Pep1 was initially characterized in *U. maydis* as an essential effector protein that targets the maize POX12, suppressing the oxidative burst and facilitating fungal invasion (Hemetsberger et al., 2015). The enhanced resistance observed in *UcPep1*-silenced plants suggests that this effector function is conserved in the *Coix-U. coicis* interaction. Silencing *UcPep1* in Coix presumably generated small interfering RNAs that were transferred to the invading fungus, suppressing *Pep1* expression and compromising its ability to counteract host defenses. This result not only validates the utility of our system for studying pathogen virulence genes but also highlights the potential of VIGS-based HIGS as a functional genomics tool for dissecting molecular dialogues in non-model pathosystems. Moreover, it suggests that transient HIGS approaches could eventually inform the development of RNA-based disease control strategies in Coix.

Overall, this study presents the first TRV-based VIGS system for *C.lacryma-jobi*. Through systematic optimization and the novel application of methylglyoxal, we achieved efficient gene silencing and demonstrated its functional relevance in host–pathogen interactions. This platform provides a foundational tool for advancing functional genomics and disease resistance research in Coix.

## Data Availability

The original contributions presented in the study are included in the article/**Supplementary Material**. Further inquiries can be directed to the corresponding authors.

## Supplementary material

Supplementary Figure 1 Phylogenetic analysis of ClPDS1 and its homologous proteins in other plants.

Supplementary Figure 2 Comparison of amino acid sequences of ZmPDS, SbPDS, and ClPDS.

Supplementary Figure 3 Schematic diagram of vacuum infiltration *Agrobacterium* process.

Supplementary Figure 4 Isolation and Mating of Haploid Strains of *Ustilago coicis*.

Supplementary Figure 5 Methylglyoxal treatment does not affect the resistance of Coix to *Ustilago coicis.*

Supplementary Figure 6 Relative expression level of *ClPDS* and *ClNPR1* in the corresponding plants (*ClEF1a* as the internal reference gene).

Supplementary Table 1 Primer sequences used in this study.

Supplementary Table 2 Ct values of the RT-qPCR experiments in this study.
