# Supplementary material for: Establishment of a TRV-VIGS system in Coix (Coix lacryma-jobi L.) for functional study of host–pathogen interactions
Source: Front Plant Sci. 2026 Apr 14;17:1816009. doi: 10.3389/fpls.2026.1816009 (PMC13121349; doi:10.3389/fpls.2026.1816009)
Supplement: Supplementary Figure 1 — Phylogenetic analysis of ClPDS1 and its homologous proteins in other plants. [file DataSheet1.pdf]

Supplementary Material

Establishment of a TRV-VIGS system in Coix (*Coix lacryma-jobi*) for functional study of host–pathogen interactions

Xiaosheng Zhao[1†*], Guocui Wang[1†], Fang He[1†], Xiang-dong Li[2], Chunli Zhou[1], Qiuping Liu[1], Leitao Tan[1], Qingbei Weng[1,3*]

Supplementary Figures

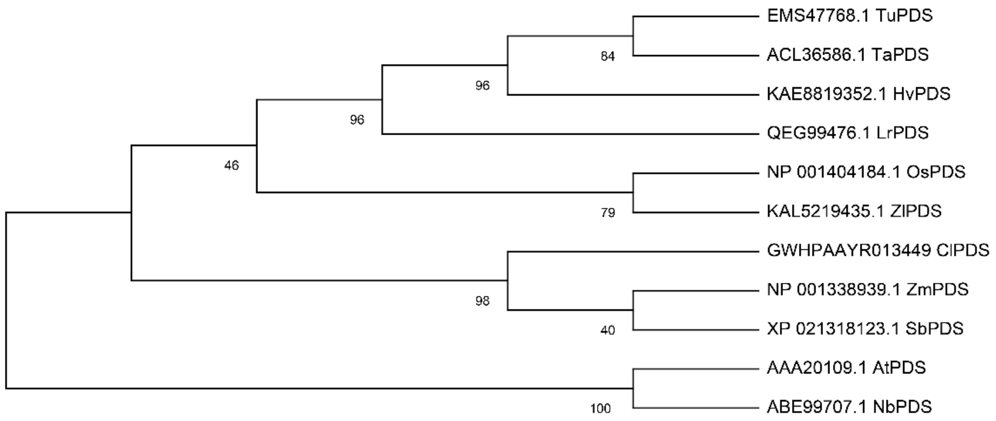

Supplementary Figure S1 Phylogenetic analysis of ClPDS1 and its homologous proteins in other plants.

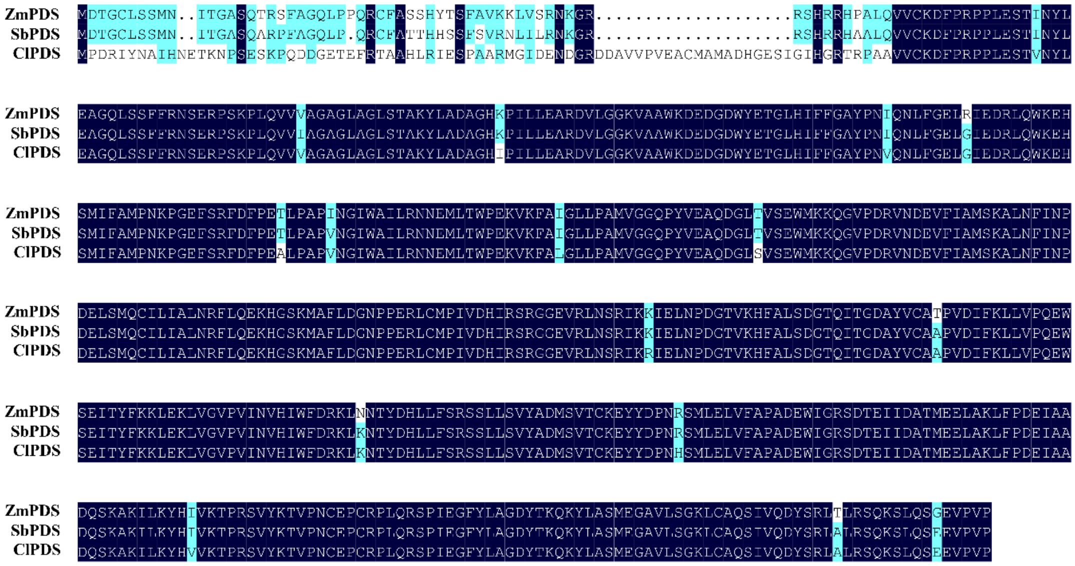

Supplementary Figure S2 Comparison of amino acid sequences of ZmPDS, SbPDS, and ClPDS.

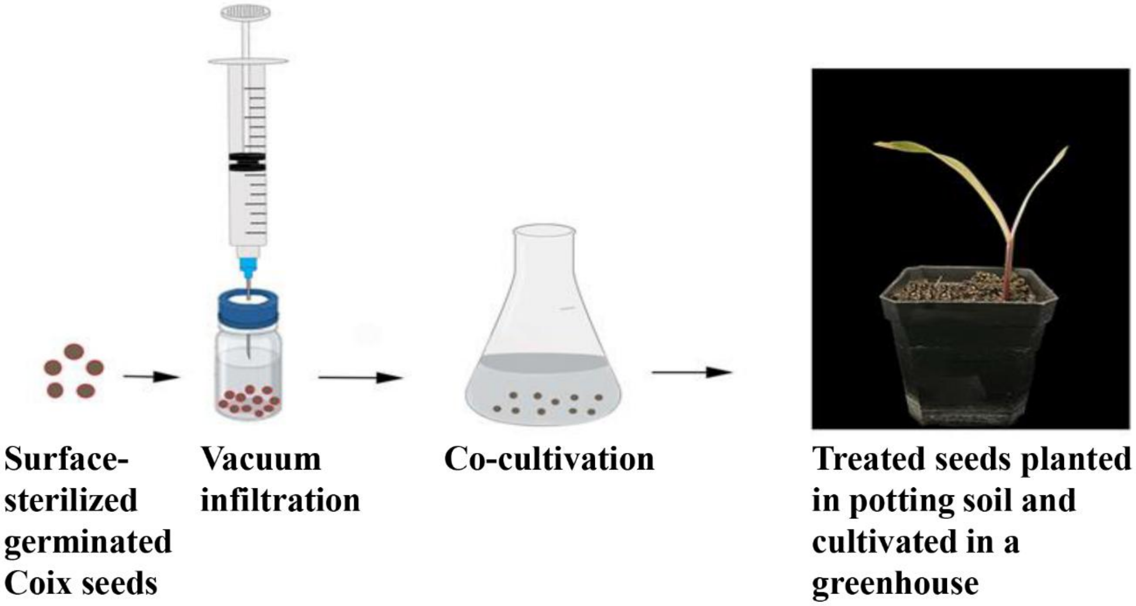

Supplementary Figure S3 Schematic diagram of vacuum infiltration *Agrobacterium* process.

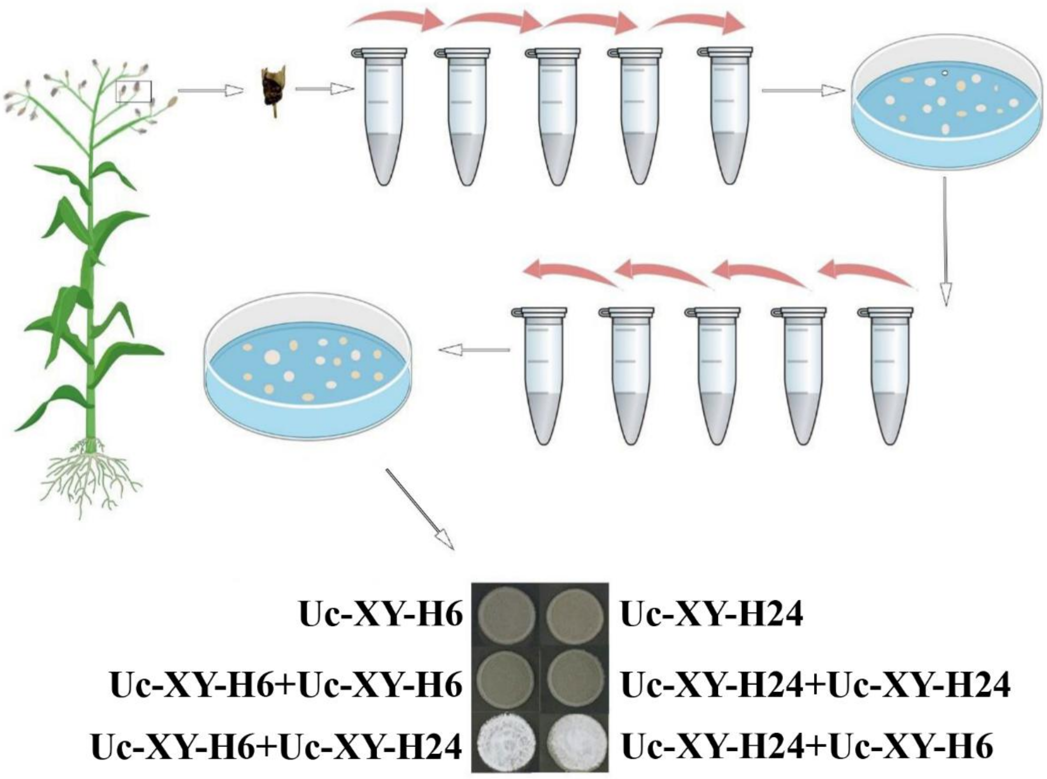

Supplementary Figure S4 Isolation and Mating of Haploid Strains of *Ustilago coicis.*

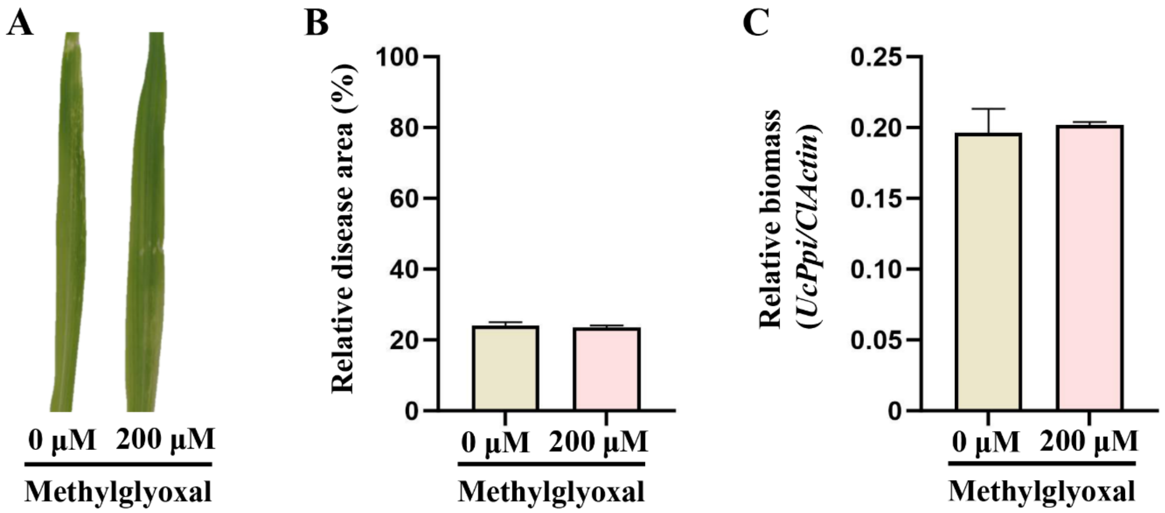

Supplementary Figure S5 Methylglyoxal treatment does not affect the resistance of coix to *Ustilago coicis.*

(A-C) Phenotypes (A), relative disease area (B), and relative fungal biomass (C) of Coix plants 4 days post-inoculation with *Ustilago coicis*.

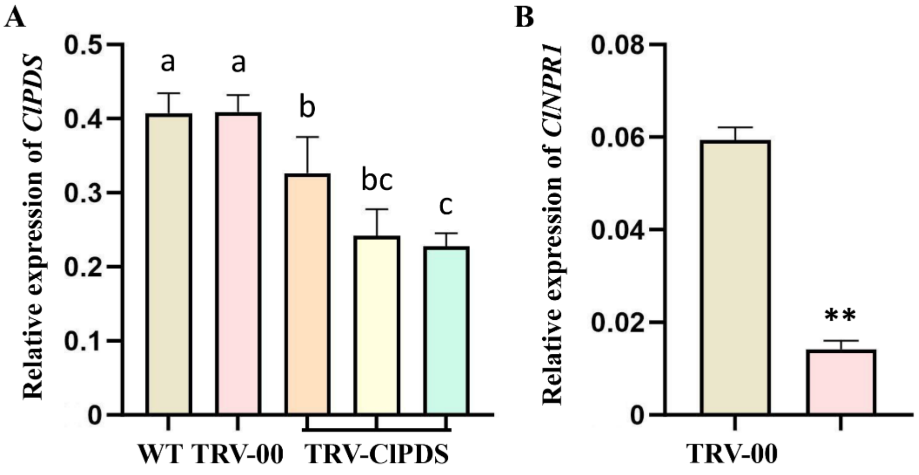

Supplementary Figure S6 Relative expression level of *ClPDS* and *ClNPR1* in the corresponding plants (*ClEF1a* as the internal reference gene).

(A) Relative expression level of *ClPDS* in plants at 15 days after TRV-VIGS-mediated silencing.

(B) Relative expression level of *ClNPR1* in TRV-VIGS-silenced *ClNPR1* (TRV-ClNPR1) plants at 4 days post-inoculation with *U. coicis.*

Data are presented as mean ± SD (n = 3). Similar results were obtained from three independent biological replicates. The expression of *ClPDS* was normalized to the *ClEF1a* gene. Statistical significance was determined using Student's *t*-test (** $p < 0.01$) and one-way ANOVA with Tukey's test (Bars with different letters are significantly different at $p < 0.05$).

Supplementary Table

Table S1. Primer sequences used in this study.

Primer name	Primer sequence (5’to 3’)
TRV-ClPDS-F	5'-ATACCATGGTCATCAGGAAACAACTTCG-3'
TRV-ClPDS-R	5'-TATGGTACCTGTTCATATATGGTTTGAC-3'
TRV-ClNPR1-F	5'-ATAGGATCCGTACAAGATGGAGGAGCT-3'
TRV-ClNPR1-R	5'-ATAGAATTCAGGTGACCTCGGTGATCT-3'
TRV-UcPep1-F	5'-ATAGGATCCAGACGTTCACCATTACCTT-3'
TRV-UcPep1-R	5'-ATAGAATTCACCGATAGGCTTGAGAGC-3'
qRT-ClPDS-F	5'-GCAGGATTATAGCAGGCTCG-3'
qRT-ClPDS-R	5'-GGACCATAACAGGTGTCTCCAA-3'
qRT-ClNPR1-F	5'-CGTCACCTCGCCTTTACTGG-3'
qRT-ClNPR1-R	5'-TTGAAGGGCAACAGGCAACC-3'
qRT-ClActin-F	5'-GTTGGATGAGATGGGTGTAGAA-3'
qRT-ClActin-R	5'-TGGAGTTGTATGTGGCTTCATG-3'
qRT-UcPep1-F	5'-TGGACCTCTCTCAACCGCAA-3'
qRT-UcPep1-R	5'-GTCTCCATCACCGCCAGAAC-3'
qRT-UcPpi- F	5'-AGGTCGTTGACGGTATGGAC-3'
qRT-UcPpi -R	5'-TTAGCACTCACCGCAGTCG-3'
qRT-ClEF1α-F	GGCTTCCAACTCCAAGGATGAC
qRT-ClEF1α-R	ATCGCCTGTCAATCTTGGTAACG
TRV-CX-F	ACATTGTTACTCAAGGAAGCAC

Table S2. Ct values of the RT-qPCR experiments in this study.

Samples	Genes	CT1	CT2	CT3
WT	Actin	23.769	23.746	23.636
	PDS	26.105	26.007	26.075
TRV-00	Actin	23.595	23.798	23.758
	PDS	25.971	25.917	26.255
TRV-PDS-1	Actin	24.367	24.583	24.440
	PDS	27.195	27.862	27.579
TRV-PDS-2	Actin	24.583	24.539	24.342
	PDS	27.993	28.170	28.011
TRV-PDS-3	Actin	24.111	24.048	23.950
	PDS	27.977	28.023	28.070
TRV-00	Actin	24.664	24.319	24.734
	NPR1	26.358	26.103	26.408
TRV-NPR1	Actin	20.432	20.371	20.403
	NPR1	24.427	24.354	24.315
WT	EF1a	22.408	22.579	22.592
	PDS	23.772	23.770	23.930
TRV-00	EF1a	22.814	22.961	22.862
	PDS	24.164	24.284	24.063
TRV-PDS-1	EF1a	22.941	23.034	22.946
	PDS	24.918	24.467	24.524
TRV-PDS-2	EF1a	22.783	22.823	22.963
	PDS	25.067	24.868	24.813
TRV-PDS-3	EF1a	23.103	22.892	22.837
	PDS	25.168	25.164	24.919
TRV-00	EF1a	19.717	19.608	19.733
	NPR1	23.780	23.625	23.881
TRV-NPR1	EF1a	18.343	18.766	18.415
	NPR1	24.669	24.720	24.582
